# Erythema Protection Efficacy of Plant-Derivative Compounds in Mice Based on Narrow-Band Reflectance Spectroscopy Data

**DOI:** 10.3390/life16010176

**Published:** 2026-01-21

**Authors:** Diego Armando Villamizar Mantilla, Luis Alberto Nuñez, Elena E. Stashenko, María Pilar Vinardell, Jorge Luis Fuentes

**Affiliations:** 1 Laboratorio de Microbiología y Mutagénesis Ambiental (LMMA), Grupo de Investigación en Microbiología y Genética, Escuela de Biología, Facultad de Ciencias, Universidad Industrial de Santander (UIS), Bucaramanga 680002, Colombia; 2 Escuela de Física, Facultad de Ciencias, Universidad Industrial de Santander, Bucaramanga 680002, Colombia; 3 Departamento de Física, Universidad de Los Andes, Mérida 5101, Venezuela; 4 Centro de Investigación en Biomoléculas, CIBIMOL, Facultad de Ciencias, Universidad Industrial de Santander (UIS), Bucaramanga 680002, Colombia; elenastashenko@gmail.com; 5 Departamento de Bioquímica y Fisiología, Facultad de Farmacia y Ciencias de la Alimentación, Universitat de Barcelona, 08028 Barcelona, Spain

**Keywords:** UVB radiation, minimal erythema dose, photoprotection, mice

## Abstract

**Background**: Plants represent an important source of photoprotective compounds that are capable of protecting human skin from solar-induced damage. In this study we investigated the suitability of a murine model for estimating the Erythema Protection Efficacy (EPE) of natural compound. **Methods**: UVB-induced skin erythema in albino BALB/c mice was quantified using a Mexameter MX18 MDD colorimeter. The ARRIVE principle was followed. The Minimum Erythema Dose (MED) was determined based on Log_10_ dose–erythema response curves. EPE values for UV filters (e.g., titanium dioxide or zinc oxide) and selected plant-derived compounds (apigenin, caffeic acid, epigallocatechin gallate, kaempferol, and pinocembrin) were calculated as the ratio between the MED of protected skin and that of unprotected skin. **Results**: The UVB-induced erythema in both female and male mouse skin followed a linear response. Erythema intensity varied by sex and by the dorsal skin area examined. MED values ranged from 39 to 57 mJ/cm^2^ in female mice and from 71 to 80 mJ/cm^2^ in male mice. In both sexes, MED increased linearly with the logarithm of the radiation dose. All tested compounds (apigenin, caffeic acid, epigallocatechin gallate, kaempferol, and pinocembrin) provided protection against UV-radiation-induced erythema in mouse skin. Among them, apigenin, caffeic acid, and kaempferol exhibited the highest EPE values, indicating strong potential for incorporation into sunscreen formulations. **Conclusions**: The murine EPE metric proved to be a useful tool for identifying plant-derived compounds with potential relevance for the photoprotection of human skin.

## 1. Introduction

Overexposure of human skin to solar radiation represents a public health problem due to its well-stablished carcinogenic effects [1,2]. Ultraviolet (UV) radiation induces the formation of cyclobutane pyrimidine dimers (CPDs), which initiate mutagenic, inflammatory, and carcinogenic processes in human skin [3]. Young et al. [4] were the first to demonstrate a direct relationship between UV-induced CPD formation and skin erythema, proposing erythema as a surrogate marker of UV-induced DNA damage. Consequently, measurements of UV-induced erythema, particularly the MED, have become essential for evaluating the efficacy of photoprotective strategies for human skin [5,6].

Phytochemicals are well-known for their antioxidant, chemopreventive, and anti-inflammatory properties as well as their ability to mitigate UV-radiation-induced skin damage [7,8]. Using a narrow-band reflectance method [9], we previously demonstrated that phytochemicals inhibit UV-induced erythema in murine skin [10]. Our study suggested that applying the narrow-band reflectance method in a murine model is an effective approach for screening and identifying photoprotective phytochemicals.

There are at least three main reasons to develop alternative approaches to the Sun Protection Factor (SPF) metric [5] for assessing the photoprotective activity of sunscreen ingredients. First, the current “gold standard” method (European Standard ISO 24444:2019), which is conducted in human volunteers [11,12], is laborious and costly because it requires specialized equipment, limiting its applicability in large-scale phytochemical screening. Second, the gold standard approach may occasionally cause skin damage in sensitive volunteers, raising ethical concerns. For similar reasons, in vivo studies of the biological effects of photoprotective compounds cannot be conducted extensively in humans. In this context, murine models represent a valuable alternative for screening molecules with anti-erythema activity under UV radiation and for mechanistic studies [10]. Third, the alternative in vitro method used to evaluate the photoprotective efficacy of sunscreens (European Standard ISO 24443:2012) uses polymethylmethacrylate (PMMA) as a substrate, which is a plastic matrix that differs substantially from human skin. Consequently, this method is not suitable for studying the photoprotection mechanisms of sunscreen ingredients, particularly those involved in preventing UV-induced DNA damage or carcinogenic processes in human skin. Such mechanisms can be reliably investigated using a mammalian skin matrix.

The present study hypothesized that a murine model could be used to establish an EPE metric analogous to the human Sun Protection Factor (SPF), thereby facilitating the screening of phytochemicals with potential applications in human skin photoprotection. According, this study aimed to characterize UVB-induced erythema kinetics in albino BALB/c mice using narrow-band reflectance methods, determine the MED in murine skin, and determine a stable EPE metric for conventional UV filters and plant-derived compounds.

## 2. Materials and Methods

### 2.1. UVB-Induced Erythema Kinetics 

We used albino BALB/c mice purchased from the Corporation for Biological Research (CIB, Medellín, Colombia). Animal maintenance, preparation for experiments, and irradiation were conducted as previously described [10]. The ARRIVE principle was followed. The kinetics of UVB-induced erythema in mice skin were studied at radiation doses of 70–200 mJ/cm^2^. Erythema was measured using a Mexameter MX18 MDD model colorimeter (Courage–Khazaka Electronic, Cologne, Germany). The Mexameter colorimeter measured absorbed and reflected light at three wavelengths: green λ = 568 nm, red λ = 660 nm, and infrared λ = 870 nm. From the quantity of emitted light, the amount of light absorbed by the skin was calculated. The hemoglobin (erythema) value was computed based on the measured back-reflected light in the green (568 nm) and red (660 nm) bands as follows [13]: hemoglobin (erythema) = 500/log5 × [log (red-reflection/green-reflection) + log5]. Measurements were performed immediately pre-irradiation (0 h) as well as 24 and 48 h after irradiation of the upper, middle, and lower back dorsal areas (1 cm^2^ each) of 36 subjects (18 female + 18 male mice). This colorimeter probe featured a spring that maintained constant pressure on the skin, enabling automatic absorbance and reflectance measurements on a 5 mm surface diameter. UVB-induced erythema in mice was computed by normalizing to untreated skin as follows: Erythema Index (EI) = reflectance of irradiated skin − reflectance of nonirradiated skin [14]. The mean EI value for each treatment (radiation dose) and corresponding standard errors were calculated (n = 3) and plotted using the ggplot2 program in R software [15]. Thus, the best erythema kinetic function was determined. The MED values in the mice were calculated graphically based on Log_10_ dose–erythemal response curves [16], using data from 36 subjects (n = 18 females and 18 males).

### 2.2. Erythema Protection Efficacy of the Compounds in Mice

We determined EPE values for standard filters (e.g., titanium dioxide and zinc oxide) and plant-derived compounds (e.g., apigenin, caffeic acid, epigallocatechin gallate (EGCG), kaempferol, and pinocembrin,) purchased from Sigma-Aldrich Co. Inc. (Milwaukee, WI, USA). The mice were grouped as follows: A, untreated mice; B, mice treated only with humectant vehicle (25% glycerin); C, mice treated with humectant and irradiated at doses between 200 and 1200 mJ/cm^2^; and D, mice treated with the humectant, compound (μg/cm^2^), and irradiated at the dose used in treatment C. All treatments were applied to the upper back of each mouse (*n* = 3). Because plant-derived compounds exhibit diverse solubility profiles, compound-specific solvents were selected for the preparation of the stock solutions [10]. Then, the stock solutions were mixed with 4 μL of pure glycerin humectant vehicle (Laboratorios León S.A., Bucaramanga, Colombia) and with distilled water until reaching a spreading volume (16 μL) and compound concentrations as follows: apigenin (48.6 μg/cm^2^), caffeic acid (80.7 μg/cm^2^), EGCG (198 μg/cm^2^), kaempferol (100 μg/cm^2^), and pinocembrin (24.5 μg/cm^2^). These concentrations were selected based on their in vitro effective photoprotection, photostability, and safety in human fibroblast cells [17]. The standard filters, titanium dioxide (500 μg/cm^2^) and zinc oxide (500 μg/cm^2^), were prepared at a concentration (25% in the mixture) recommended for human photoprotection [18]. The Erythema Inhibition Percentage (EIP) was computed for each cotreatment as follows: EIP = 1 − (EI_p_/EI_u_) × 100, where EI_p_ is the EI value of protected mice skin, and EI_u_ is the EI value of unprotected mice skin.

Based on the SPF metric [12], we computed EPE values for each compound as follows: EPE = $\frac{{MED}_{p}}{{MED}_{u}}$, where MED_p_ is the minimal erythemal dose of an individual subject’s protected skin (compound tested), and MED_u_ is the minimal erythemal dose of an individual subject’s unprotected skin (humectant vehicle + UVB radiation). Since incomplete or partial photoprotection can occur at doses higher than the MED_p_ (see Figure S1), we computed a corrected EPE index considering the area under the curve of the incomplete protection zone as follows: EPE_c_ = EPE + $\left(\frac{D_{max}}{{MED}_{p\;}}\right)\left(\frac{\int_{{MED}_{u\;}}^{SED}\;dD\left(g(D)-E_{0}\right)+\int_{SED}^{{MED}_{p\;}}\;dD\left(E_{1}-E_{0}\right)}{\int_{{MED}_{p\;}}^{D_{max}}\;dD\left(E_{1}-f(D)\right)}\right)$, where EPE is the erythema protective efficacy of the sample as indicated above, D_max_ is the maximum dose to the incompletely protected zone, SED is the saturation erythema dose delivered to the completely protected zone, E_0_ and E_1_ are the erythema indices (EI) at MED_u_ and MED_p_, respectively, and g(D) and f(D) are the erythema index values as a function of dose.

### 2.3. Statistical Analysis

The average IE, MED, EPE, and EPE_C_ values, along with their corresponding standard errors, were calculated. The data passed the Kolmogorov–Smirnov and F-maximum tests for normality and variance homogeneity, respectively. Therefore, the groups were subsequently compared using the parametric Tukey’s test. A Pearson’s product–moment correlation analysis was used to estimate relation between radiation dose and EI estimates, as well as between EPE or EPE_C_ and the expected human SPF estimates. For all statistical analyses, a *p*-value < 0.05 indicated statistical significance. The R platform [15] was used for all analyses.

## 3. Results

### 3.1. UVB-Induced Skin Erythema in Albino BALB/c Mice

**Figure 1:**
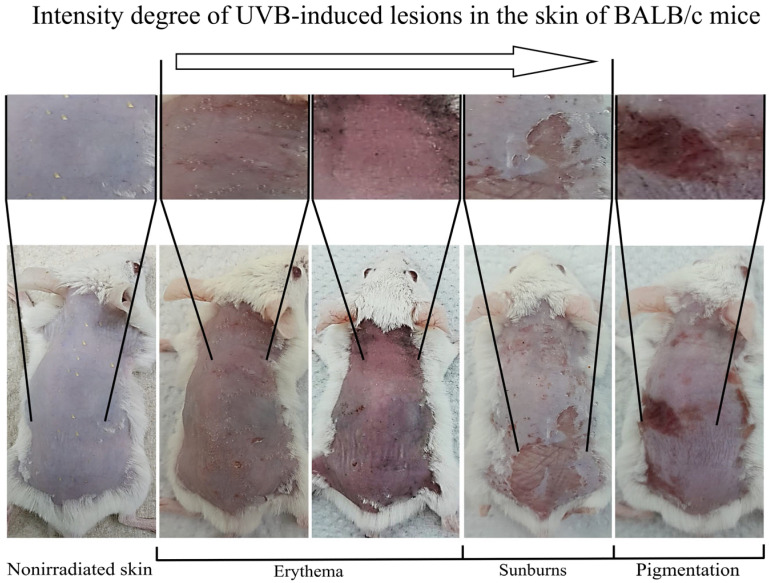
Different UVB-induced lesions in the skin of BALB/c mice.

UVB-induced skin damage in BALB/c mice was studied (Figure 1). Visible skin damage in UVB-irradiated mice was classified as follows: female: erythema (71%), burns (20%), pigmentation (9%); and males: erythema (62%), burns (30%), and pigmentation (8%).

The degree of UVB-induced erythema was measured using narrow-band reflectance spectroscopy (Table 1). Skin reflectance increased from pre-irradiation (0 h) to 48 h post-irradiation. The EI values of the mouse skin depended on radiation dose (R = 0.79–0.98, *p* ˂ 0.05), and these were consistently higher in females than in males. Additionally, the EI values varied, with the EI in back dorsal area being higher in upper areas.

**Figure 2:**
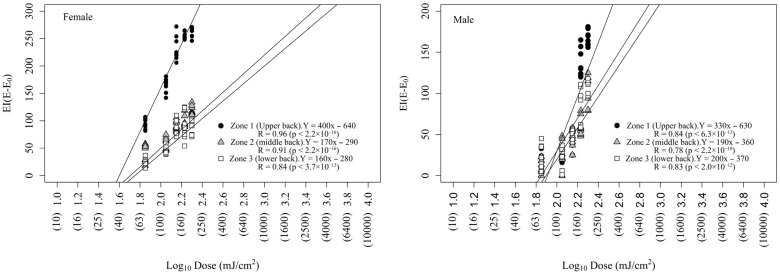
Pooled dose–response curves to UVB radiation obtained from a total of 42 subjects (n = 21 female + 21 male).

A linear graphical method based on Log_10_ dose–erythemal response curves permitted the estimation of the threshold dose (*X*-axis intercept) or MED (Figure 2).

The erythema dose–response curves fit a linear function, showing the best fit (R = 0.84–0.96, *p* < 6.3 × 10^−13^) for the upper back dorsal area. The MED values varied depending on sex and dorsal region as follows: females: upper back (39 mJ/cm^2^) < central back (51 mJ/cm^2^) < lower back (57 mJ/cm^2^); males: lower back (71 mJ/cm^2^) < central back (77 mJ/cm^2^) < upper back (80 mJ/cm^2^). This confirmed that female mice were more sensitive to UVB radiation than male mice.

### 3.2. Erythema Protection Efficacy of Phytochemicals

To determine the EPE values for each compound, we studied UVB-induced erythema in the presence and absence of plant-derived compounds on the upper back area, which showed the best linear function fit (see Figure 2) in both female (R = 0.96, *p* < 2.2 × 10^−16^) and male (R = 0.84, *p* < 6.3 × 10^−16^) mice.

Table 2 presents the reflectance and EI values of the different treatments. For all treatments, reflectance values increased from the time of irradiation (0 h) to 48 h post-irradiation in both female and male mice. Exposure to 200 mJ/cm^2^ of UVB radiation increased the EI values relative to the negative control. Cotreatment with glycerin (25%) + UVB radiation (200 mJ/cm^2^) also increased the EI values compared with the negative control, although this increase was slightly lower than that observed in UVB-irradiated (200 mJ/cm^2^) mouse skin. Because glycerin was used as an emollient to facilitate the application of all treatments to mouse skin, the EI value obtained in the cotreatment with glycerin + UVB radiation (200 mJ/cm^2^) (highlighted in bold) was used as the reference control for comparison in phytochemical-UVB cotreatment experiments.

Cotreatment with apigenin (48.6 µg/cm^2^), caffeic acid (448 µM/cm^2^), EGCG (431 µM/cm^2^), and kaempferol (349 µM/cm^2^) significantly reduced UVB-induced erythema and showed a relevant EIP value (EIP ≥ 50%) at UVB doses of 400, 800, 400, and 800 mJ/cm^2^, respectively. Pinocembrin (95.61 µM/cm^2^) significantly reduced the induced erythema at a UVB dose of 400 mJ/cm^2^ and achieved a relevant EIP value at 200 mJ/cm^2^.

We calculated the EPE metric to quantify the effectiveness of the tested compounds (Table 3). Based on the EPE values, the relative photoprotective efficacy ranked as follows: kaempferol (20.5 ± 0.0) > apigenin = titanium dioxide = zinc oxide (15.4 ± 0.0) > caffeic acid (10.0 ± 0.0) > pinocembrin (5.0 ± 0.0) > EGCG (2.5 ± 0.0). These results demonstrated that low doses of kaempferol (100 µg/cm^2^) and apigenin (48.6 µg/cm^2^) yielded EPE values higher than, or similar to, those obtained with conventional inorganic filters (titanium dioxide or zinc oxide) applied at 500 µg/cm^2^. These results support the potential utility of the evaluated phytochemicals as UV filters in sunscreen formulations.

Because incomplete photoprotection may occur at doses higher than the MED_p_ (Figure S1), corrected EPE (EPE_c_) values were calculated for each compound (Table 3). The corrected ranking was as follows: kaempferol (23.4 ± 0.0) ˃ titanium dioxide = zinc oxide (20.1 ± 0.0) ˃ apigenin (19.9 ± 0.0) ˃ caffeic acid (13.4 ± 0.0) ˃ pinocembrin (7.6 ± 0.0) ˃ EGCG (3.4 ± 0.0). Table 3 also presents the predicted SPF values (SPF = MED_p_/human MED) for each photoprotective compound for each human phototype (I–IV). The Pearson’s product–moment correlation analysis indicated moderate to high correlations between EPE or EPE_C_ and expected human SPF estimates. These predictions suggest that apigenin, caffeic acid, and kaempferol can be used not only as antioxidants but also as effective UV-filter ingredients in sunscreen formulations. Our results support the potential of these plant-derived compounds in human skin photoprotection.

## 4. Discussion

This study describes the erythema dose–response kinetics in mouse skin following exposure to artificial UVB radiation. The observed kinetics were comparable to those previously reported for human skin [20,21]. The Log_10_ dose–erythema response curves enabled the calculation of the threshold dose, defined as the X-axis intercept or MED_u_, using an approach analogous, to that applied in human studies [16,22]. The MED_u_ values obtained in BALB/c mice (39–80 mJ/cm^2^) were slightly lower but comparable to those reported for visually scored erythema in mouse skin (41–92 mJ/cm^2^) [23]. Our results demonstrated that UVB-induced erythema was more pronounced in female mice than in male mice and varied with the dorsal skin area assayed. These differences may be associated with regional variations in skin vascularization, as enhanced vascularization has previously been correlated with increased erythema responses in mice [24]. Consistent with published reports, MED_u_ values are known to vary with skin phototype in humans [19,21,25] and genetic background in mice [23].

Despite the increasing interest in plant species as sources of photoprotective agents [7,8,26], only a limited number of phytochemicals, such as caffeic, ferulic, and chlorogenic acids, and EGCG, have demonstrated photoprotective efficacy in vivo [27,28,29,30,31]. In the present study, we report EPE values for several phytochemicals (apigenin, caffeic acid, EGCG, kaempferol, and pinocembrin) that have previously exhibited photoprotective activity in vitro [32,33]. Our results indicate that these compounds reduce UVB-induced skin erythema in vivo in a manner comparable to that of established inorganic UV filters, such as titanium dioxide and zinc oxide. As reported in a previous study [34], our data also highlight the critical role of solvents in photoprotection. For instance, EGCG exhibited low protection (EPE = 2.5 ± 0.0), despite prior evidence of its in vivo photoprotective activity [27,29]. This discrepancy may be attributed to differences in the polarity of the solvent and vehicle composition (acetone and glycerin), which can affect compound solubility, skin distribution, and uniformity of application, thereby influencing photoprotection measurements.

Our results are consistent with previous reports on the skin-protective properties of these phytochemicals, including anti-inflammatory and chemopreventive effects [see Table S1]. Previous studies [35,36] have shown that pinocembrin and kaempferol exhibit antigenotoxic activity against UVB radiation in human embryo kidney and fibroblast cells. In addition, kaempferol has been reported to promote DNA damage repair in mouse skin after UV irradiation. These antigenotoxic effects may be attributed to mechanisms such as the inhibition of cyclobutene pyrimidine dimer (CPD) formation, enhanced CPD removal, and the restoration of normal cell cycle progression. All these findings highlight the strong potential of these phytochemicals for cosmetic and sunscreen applications. Furthermore, we have recently demonstrated that several plant species, including *Posoqueria latifolia*, and *Rosa centifolia*, represent natural sources of bioactive phytochemicals [37]. However, the development of cost-effective isolation processes will be essential to ensure a stable and sustainable supply of these raw materials.

The narrow-band reflectance method has been proven useful for the assessment of multiple dermatological conditions in humans [9,13,38]. In the present study, we demonstrate that the Mexameter MX18 colorimeter (Courage–Khazaka Electronic, Cologne, Germany) produces reproducible results, providing, a cost-effective alternative approach for estimating photoprotective efficacy during early-stage photoprotective compound screening. The EPE metric also correlated to expected human SPF estimates, especially SPF_C_ metric. Because the EPE index is based on in vivo erythema measurements, compounds identified using this approach are expected to be relevant for human photoprotection.

## 5. Conclusions

UVB-induced erythema in mouse skin exhibits a linear kinetic response up to a dose of 200 mJ/cm^2^. The MED_u_ values obtained from BALB/c mice (39–80 mJ/cm^2^) were slightly lower but comparable to those previously reported for visually scored erythema in mouse skin (41–92 mJ/cm^2^). Plant-derived compounds including apigenin, caffeic acid, EGCG, kaempferol, and pinocembrin showed photoprotective effects in mice, with apigenin, caffeic acid, and kaempferol being the most promising candidates for inclusion in sunscreen formulations. Quantitative erythema measurements using mice facilitate the calculation of EPE values during the bioprospecting of plant-derived UV-filter compounds. The EPE metric, particularly the SPF_C_ estimate, correlated with the expected human SPF values for the compounds studied. Therefore, the EPE_C_ metric shows potential for predicting human photoprotection.

## Figures and Tables

**Table 1:** Reflectance measurements before irradiation (t = 0 h) and after irradiation (t = 24 and 48 h) of different dorsal areas of female and male mouse skin. The EI values were calculated as follows: EI = reflectance in irradiated skin − reflectance in nonirradiated skin (in bold values). The average values and the corresponding standard errors, calculated from at least three animals (n = 3), are given.

Radiation	Weight	Reflectance and Erythema Measurements in Mouse Skin											
Doses		Upper Back Dorsal Areas				Middle Back Dorsal Areas				Lower Back Dorsal Areas			
(mJ/cm^2^)	(g)	t (0 h)	t (24 h)	t (48 h)	EI ^†^	t (0 h)	t (24 h)	t (48 h)	EI ^†^	t (0 h)	t (24 h)	t (48 h)	EI ^†^
	Female												
0	18.3 ± 1.2	**255 ± 0**	267 ± 2	270 ± 0	15 ± 3	**178 ± 2**	180 ± 1	185 ± 2	7 ± 1	**161 ± 2**	162 ± 2	161 ± 2	0 ± 0
70	18.1 ± 7.4	260 ± 1	283 ± 3	349 ± 3	94 ± 3	177 ± 1	180 ± 3	219 ± 4	41 ± 5	157 ± 1	162 ± 2	186 ± 1	25 ± 3
112	18.2 ± 0.9	268 ± 4	296 ± 3	418 ± 5	163 ± 5	176 ± 2	204 ± 3	235 ± 4	57 ± 4	160 ± 2	181 ± 3	207 ± 3	46 ± 2
140	19.0 ± 0.6	272 ± 5	326 ± 3	485 ± 4	230 ± 7	188 ± 1	210 ± 4	268 ± 3	91 ± 3	164 ± 2	193 ± 3	245 ± 3	84 ± 4
168	18.2 ± 0.8	275 ± 1	335 ± 2	512 ± 2	257 ± 2	184 ± 3	224 ± 6	275 ± 3	98 ± 3	164 ± 5	212 ± 3	253 ± 7	92 ± 9
200	17.2 ± 0.8	263 ± 2	306 ± 7	501 ± 4	246 ± 5	186 ± 1	217 ± 7	309 ± 7	131 ± 8	159 ± 2	207 ± 8	271 ± 9	110 ± 9
					(R = 0.97 *)				(R = 0.98 *)				(R = 0.98 *)
	Male												
0	22.1 ± 0.5	**157 ± 3**	160 ± 3	175 ± 4	18 ± 5	**103 ± 5**	109 ± 7	115 ± 11	12 ± 6	**66 ± 3**	72 ± 5	79 ± 5	13 ± 3
70	22.9 ± 1.3	140 ± 5	176 ± 6	178 ± 2	21 ± 4	80 ± 2	97 ± 4	103 ± 4	0 ± 0	67 ± 7	88 ± 3	86 ± 2	20 ± 5
112	24.3 ± 1.2	160 ± 5	171 ± 8	182 ± 3	25 ± 2	83 ± 4	97 ± 4	121 ± 4	18 ± 6	73 ± 7	89 ± 5	89 ± 4	23± 5
140	23.1 ± 0.7	163 ± 4	187 ± 1	202 ± 4	45 ± 3	95 ± 2	105 ± 2	126 ± 3	23 ± 5	80 ± 4	102 ± 1	112 ± 1	46 ± 2
168	22.3 ± 1.3	158 ± 2	216 ± 3	294 ± 4	137 ± 5	101 ± 5	121 ± 5	161 ± 3	58 ± 4	66 ± 3	78 ± 5	154 ± 10	88 ± 7
200	21.0 ± 1.0	166 ± 1	294 ± 4	326 ± 2	169 ± 3	96 ± 4	175 ± 3	207 ± 2	104 ± 7	86 ± 1	114 ± 1	175 ± 2	109 ± 3
					(R = 0.82 *)				(R = 0.79 *)				(R = 0.87 *)

**^†^**, The EI values were calculated using reflectance measurements obtained 48 h post-irradiation [14]. *, Significant (*p* ˂ 0.05) correlation between radiation dose and erythema index (EI) values.

**Table 2:** Reflectance measurements pre-irradiation (t = 0 h) and post-irradiation (t = 24 and 48 h) of upper back dorsal skin area of female mice. The EI values were calculated as follows: EI = reflectance in irradiated skin (t = 48 h) − reflectance in nonirradiated skin (t = 0 h). The average values and the corresponding standard errors were calculated from at least three animals (n = 3). The EIP values (see Section 2) are also given.

Treatments	Mice Weight	Reflectance and Erythema Measurements of Mice Skin				EIP
	(g)	t (0 h)	t (24 h)	t (48 h)	EI	
						
Female mice						
Untreated (negative control)	18.3 ± 1.2	255 ± 3	267 ± 2	270 ± 2	15 ± 3	-
25% Glycerin (Gly)	15.6 ± 0.3	262 ± 2	262 ± 2	266 ± 2	13 ± 3	-
UVB radiation (200 mJ/cm^2^)	18.8 ± 1.2	267 ± 2	306 ± 7	515 ± 3	260 ± 3	-
Gly + UVB radiation (200 mJ/cm^2^)	17.6 ± 0.4	252 ± 2	301 ± 2	489 ± 6	**234 ± 7**	-
						
Male mice						
Untreated (negative control)	22.9 ± 1.2	140 ± 5	160 ± 3	175 ± 4	17 ± 5	-
25% Glycerin (Gly)	20.0 ± 1.4	164 ± 2	169 ± 2	167 ± 2	1 ± 0	-
UVB radiation (200 mJ/cm^2^)	20.1 ± 2.4	166 ± 1	266 ± 5	326 ± 4	168 ± 4	-
Gly + UVB radiation (200 mJ/cm^2^)	24.5 ± 0.1	171 ± 2	274 ± 2	337 ± 3	**166 ± 3**	-
						
Apigenin (evaluated in female mice)						
Gly + apigenin (48.6 µg/cm^2^)	19.7 ± 0.9	264 ± 2	265 ± 1	266 ± 1	11 ± 4	95.3
Gly + apigenin + 200 mJ/cm^2^	17.6 ± 0.9	265 ± 2	302 ± 4	318 ± 3	63 ± 4 *	73.1
Gly + apigenin + 400 mJ/cm^2^	20.5 ± 0.4	265 ± 2	320 ± 2	316 ± 2	61 ± 2 *	73.9
Gly + apigenin + 600 mJ/cm^2^	18.2 ± 0.7	263 ± 1	414 ± 5	510 ± 2	234 ± 2 n.s.	0.0
						
Caffeic acid (evaluated in male mice)						
Gly + caffeic acid (80.7 μg/cm^2^)	22.8 ± 1.4	161 ± 1	165 ± 1	167 ± 2	1 ± 1	99.4
Gly + caffeic acid + 200 mJ/cm^2^	22.1 ± 0.4	155 ± 1	191 ± 3	219 ± 3	47 ± 5 *	71.7
Gly + caffeic acid + 600 mJ/cm^2^	23.8 ± 0.8	161 ± 2	209 ± 5	220 ± 1	49 ± 2 *	70.5
Gly + caffeic acid + 800 mJ/cm^2^	22.6 ± 0.6	161 ± 2	208 ± 1	221 ± 2	50 ± 2 *	69.9
Gly + caffeic acid + 1000 mJ/cm^2^	22.1 ± 1.0	160 ± 2	224 ± 2	284 ± 2	113 ± 1 n.s.	0.0
						
EGCG (evaluated in male mice)						
Gly + EGCG (198 µg/cm^2^)	21.1 ± 0.5	163 ± 1	167 ± 2	162 ± 3	1 ± 1	99.4
Gly + EGCG + 200 mJ/cm^2^	22.9 ± 0.6	167 ± 2	184 ± 2	204 ± 2	32 ± 2 *	63.8
Gly + EGCG + 400 mJ/cm^2^	22.8 ± 0.5	158 ± 2	219 ± 4	268 ± 3	97 ± 5 *	0.0
Gly + EGCG + 600 mJ/cm^2^	21.3 ± 0.3	164 ± 2	258 ± 4	336 ± 3	165 ± 5 n.s.	0.0
						
Kaempferol (evaluated in female mice)						
Gly + kaempferol (100 μg/cm^2^)	19.2 ± 0.1	264 ± 2	268 ± 3	290 ± 3	35 ± 3	85.0
Gly + kaempferol + 200 mJ/cm^2^	18.9 ± 0.1	269 ± 1	287 ± 4	309 ± 1	54 ± 4 *	76.9
Gly + kaempferol + 400 mJ/cm^2^	17.5 ± 0.2	260 ± 2	300 ± 3	319 ± 3	64 ± 5 *	72.6
Gly + kaempferol + 800 mJ/cm^2^	22.7 ± 2.8	267 ± 2	345 ± 7	360 ± 4	105 ± 5 *	55.1
Gly + kaempferol + 1000 mJ/cm^2^	17.6 ± 0.4	252 ± 2	301 ± 2	489 ± 6	234 ± 7 n.s.	0.0
						
Pinocembrin (evaluated in male mice)						
Gly + pinocembrin (24.5 µg/cm^2^)	18.6 ± 0.2	168 ± 3	163 ± 1	165 ± 2	3 ± 1	98.2
Gly + pinocembrin + 200 mJ/cm^2^	23.8 ± 0.3	165 ± 1	183 ± 3	185 ± 3	14 ± 4 *	91.6
Gly + pinocembrin + 400 mJ/cm^2^	23.3 ± 0.9	163 ± 2	209 ± 6	219 ± 4	47 ± 6 *	66.4
Gly + pinocembrin + 800 mJ/cm^2^	20.8 ± 1.3	161 ± 3	271 ± 1	302 ± 3	140 ± 3 n.s.	0.0
						
Titanium dioxide (evaluated in female mice)						
Gly + titanium dioxide (500 µg/cm^2^)	16.9 ± 1.0	259 ± 1	259 ± 1	261 ± 2	7 ± 4	-
Gly + titanium dioxide + 200 mJ/cm^2^	17.4 ± 0.4	256 ± 1	309 ± 3	320 ± 2	65 ± 4 *	72.2
Gly + titanium dioxide + 600 mJ/cm^2^	21.2 ± 3.2	260 ± 2	313 ± 3	318 ± 2	63 ± 3 *	73.1
Gly + titanium dioxide + 800 mJ/cm^2^	17.4 ± 3.6	257 ± 2	326 ± 4	445 ± 2	190 ± 4 *	18.8
Gly + titanium dioxide + 1000 mJ/cm^2^	21.6 ± 1.7	261 ± 2	328 ± 3	503 ± 3	248 ± 6 n.s.	0.0
Zinc oxide (evaluated in female mice)						
Gly + zinc oxide (500 µg/cm^2^)	19.3 ± 0.1	260 ± 2	258 ± 1	259 ± 2	6 ± 3	97.4
Gly + zinc oxide + 200 mJ/cm^2^	19.0 ± 0.3	258 ± 2	296 ± 2	302 ± 2	47 ± 2 *	79.9
Gly + zinc oxide + 600 mJ/cm^2^	18.8 ± 1.3	258 ± 2	300 ± 3	315 ± 3	60 ± 5 *	74.3
Gly + zinc oxide + 800 mJ/cm^2^	18.1 ± 0.5	255 ± 1	326 ± 4	420 ± 4	165 ± 3 *	29.5
Gly + zinc oxide + 1000 mJ/cm^2^	17.8 ± 0.5	256 ± 2	336 ± 3	469 ± 4	260 ± 2 n.s.	0.0

*, Significantly lower (*p* ˂ 0.05) EI values than in treatment with Gly + UVB radiation (200 mJ/cm^2^). n.s., non-significant inhibition.

**Table 3:** Minimal erythema doses of protected (MED_p_) and unprotected (MED_u_) mice. The erythema protective efficacy (EPE and EPE_C_) values for each treatment are also given. For each treatment, the expected SPF values (MED_p_/human MED_u_) for each human phototype (I–IV) are also shown.

Tested Samples (Mass) ^†^	SPF_in vitro_ *	MED_u_	MED_p_	EPE	EPE_C_	Expected SPF Values for Human Phototypes			
		(mJ/cm^2^)	(mJ/cm^2^)			Type I	Type II	Type III	Type IV
Glycerin 25% (solvent)	–	39–80	39–80	1.0 ± 0.0	1.0 ± 0.0	-	-	-	-
Apigenin (48.6 µg)	11 ± 0	39	400	15.4 ± 0.0	19.9 ± 0.0	11.4 ± 0.0	7.1 ± 0.0	5.7 ± 0.0	4.8 ± 0.0
Caffeic acid (80.7 µg)	36 ± 1	80	800	10.0 ± 0.0	13.4 ± 0.0	22.8 ± 0.0	14.3 ± 0.0	5.7 ± 0.0	4.8 ± 0.0
EGCG (198 µg)	19 ± 0	80	200	2.5 ± 0.0	3.4 ± 0.0	5.7 ± 0.0	3.6 ± 0.0	2.8 ± 0.0	2.4 ± 0.0
Kaempferol (100 µg)	13 ± 0	39	800	20.5 ± 0.0	23.4 ± 0.0	11.4 ± 0.0	7.1 ± 0.0	5.7 ± 0.0	4.8 ± 0.0
Pinocembrin (24.5 µg)	9 ± 0	80	400	5.0 ± 0.0	7.6 ± 0.0	11.4 ± 0.0	7.1 ± 0.0	5.7 ± 0.0	4.8 ± 0.0
Titanium dioxide (500 µg) ^‡^	40 ± 0	39	600	15.4 ± 0.0	20.1 ± 0.0	17.1 ± 0.0	10.7 ± 0.0	8.6 ± 0.0	7.1 ± 0.0
Zinc oxide (500 µg) ^‡^	40 ± 0	39	600	15.4 ± 0.0	20.1 ± 0.0	17.1 ± 0.0	10.7 ± 0.0	8.6 ± 0.0	7.1 ± 0.0
						R_1_ = 0.56 (*p* ˂ 0.14)	R_1_ = 0.55 (*p* ˂ 0.15)	R_1_ = 0.75 (*p* ˂ 0.03)	R_1_ = 0.75 (*p* ˂ 0.03)
						R_2_ = 0.63 (*p* ˂ 0.09)	R_2_ = 0.62 (*p* ˂ 0.10)	R_2_ = 0.81 (*p* ˂ 0.01)	R_2_ = 0.81 (*p* ˂ 0.01)

**^†^**, Mass of compounds that were not cytotoxic to human fibroblast cells [17]. ^‡^, Mass of standard UV filter used for sunscreen preparation based on FDA guidelines [18]. *, SPF_in vitro_ values were previously reported for each compound [17]. The MDE values for each human skin phototype were as follows: type I (35 mJ/cm^2^), type II (56 mJ/cm^2^), type III (70 mJ/cm^2^), and type IV (84 mJ/cm^2^) [19]. R_1_, Pearson’s product–moment correlation coefficient between EPE and SPF estimates calculated using R platform [15]. R_2_, Pearson’s product-moment correlation coefficient between EPE_C_ and SPF estimates using R platform [15].

## Data Availability

All data, tables, and figures are original.

## Supplementary Materials

The following supporting information can be downloaded at: https://www.mdpi.com/article/10.3390/life16010176/s1, Figure S1: Protection scenario in mice for evaluated compounds. We indicated doses and erythema index values relevant to calculated EPE index in mice as described in material and methods: MED is the minimal erythema dose, Dmax is the maximum doses to the incomplete protection zone, SED is the saturation erythema doses to the complete protection zone, E0 and E1 are the erythema indexes (EI) values at MEDu and MEDp, respectively, and g(D) and f(D) are the erythema index values as a dose function; Table S1: Previous studies that have reported protection of the studied plant compounds against UV-induced skin injuries in mammalian models. Effective concentrations of the studied compounds were given.

## Abbreviations

The following abbreviations are used in this manuscript:

CPDs	Cyclobutane pyrimidine dimers
EGCG	Epigallocatechin gallate
EI	Erythema Index
EI_p_	EI of protected mice skin
EI_u_	EI of unprotected mice skin
EIP	Erythema Inhibition Percentage
EPE	Erythema Protection Efficacy
EPE_c_	Corrected EPE
MED	Minimum Erythema Dose
MED_p_	MED of protected skin
MED_u_	MED of unprotected skin
SPF	Sun Protection Factor
