# Supplementary material for: Erythema Protection Efficacy of Plant-Derivative Compounds in Mice Based on Narrow-Band Reflectance Spectroscopy Data
Source: Life (Basel). 2026 Jan 21;16(1):176. doi: 10.3390/life16010176 (PMC12842781; doi:10.3390/life16010176)
Supplement: Supplementary file 1 [file life-16-00176-s001.zip › life-4091101-supplementary.pdf]

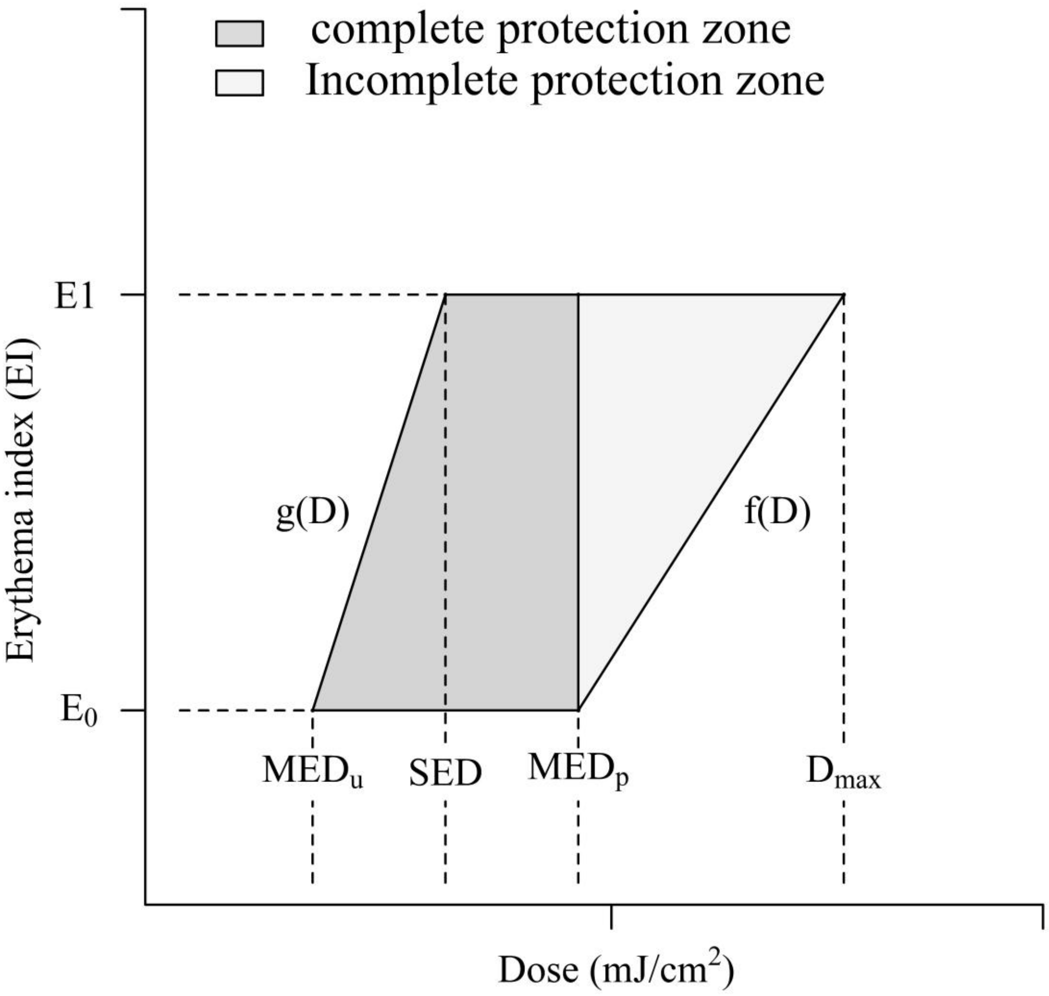

Figure S1. Protection scenario in mice for evaluated compounds. We indicated doses and erythema index values relevant to calculated EPE index in mice as described in material and methods: MED is the minimal erythema dose, D_{max} is the maximum doses to the incomplete protection zone, SED is the saturation erythema doses to the complete protection zone, E_0 and E_1 are the erythema indexes (EI) values at MED_u and MED_p, respectively, and g(D) and f(D) are the erythema index values as a dose function.

Table **S1**. Previous studies that have reported protection of the studied plant compounds against UV-induced skin injuries in mammalian models. Effective concentrations of the studied compounds were given.

**Plant compounds†**	**Relevant compound properties to human photoprotection [Sources]**
Apigenin	*In vivo* UVB-induced skin inflammation and carcinogenesis inhibition in mice [1,2]<br>*In vitro* photoprotective efficacy (SPF) against UVB radiation [3]
Caffeic acid	*In vivo* UVB-induced skin inflammation and carcinogenesis inhibition in mice [4]<br>*In vivo* photoprotective (anti-erythema) effect against UVB radiation [5]<br>*In vitro* photoprotective efficacy (SPF) against UVB radiation [3]
EGCG	*In vivo* UVB-induced immunosuppression and carcinogenesis inhibition in mice [6,7,8]<br>*In vivo* UVB-induced skin carcinogenesis inhibition in mice [9]<br>*In vivo* photoprotective (anti-erythema) effect against UVB radiation [10]
Kaempferol	*In vivo* UVB-induced skin inflammation and carcinogenesis inhibition in mice [11]<br>*In vitro* photoprotective efficacy (SPF) against UVB radiation [3]
Pinocembrin	*In vitro* photoprotective efficacy (SPF) against UVB radiation [3]

[1] Birt et al. 1997. Anticancer Res. 17:85-92. [2] Mirzoeva et al. 2018. Neoplasia. 20(9):930–942. [3] Stevanato et al. 2014. Regul Toxicol Pharmacol. 69(1):71–77. [4] Balupillai et al. 2015. Photochem Photobiol. 91(6):1458–1468. [5] Saija et al. 2000. Int J Pharm. 199(1):39–47. [6] Katiyar and Mukhtar 2011. J Leukoc Biol. 69:719–726. [7] Mittal et al. 2003. Neoplasia. 5(6):555–565. [8] Meeran et al. 2006. Cancer prevention. Clin Cancer Res. 12(7):2272–2280. [9] Lu et al. 2002. Proc Natl Acad Sci USA. 99(19):12455–12460. [10] Kim et al. 2001. Skin Pharmacol Appl Skin Physiol. 14(1):11–19. [11] Lee et al. 2010. Biochem pharmacol. 80(12):2042-2049.
